# Thiol-ene Enabled Chemical Synthesis of Truncated *S*-Lipidated Teixobactin Analogs

**DOI:** 10.3389/fchem.2020.00568

**Published:** 2020-08-04

**Authors:** Victor V. Yim, Alan J. Cameron, Iman Kavianinia, Paul W. R. Harris, Margaret A. Brimble

**Affiliations:** ^1^School of Biological Sciences, University of Auckland, Auckland, New Zealand; ^2^School of Chemical Sciences, University of Auckland, Auckland, New Zealand; ^3^Maurice Wilkins Centre for Molecular Biodiscovery, University of Auckland, Auckland, New Zealand

**Keywords:** teixobactin, lipopeptide, modified CLipPA, antimicrobial peptide, AMP, thiol-ene, lipidation

## Abstract

Herein is described the introduction of lipid moieties onto a simplified teixobactin pharmacophore using a modified Cysteine Lipidation on a Peptide or Amino acid (CLipPA) technique, whereby cysteine was substituted for 3-mercaptopropionic acid (3-MPA). A truncated teixobactin analog was prepared with the requisite thiol handle, thus enabling an array of vinyl esters to be conveniently conjugated onto the simplified teixobactin pharmacophore to yield *S*-lipidated cyclic lipopeptides.

## Introduction

**Figure 1:**
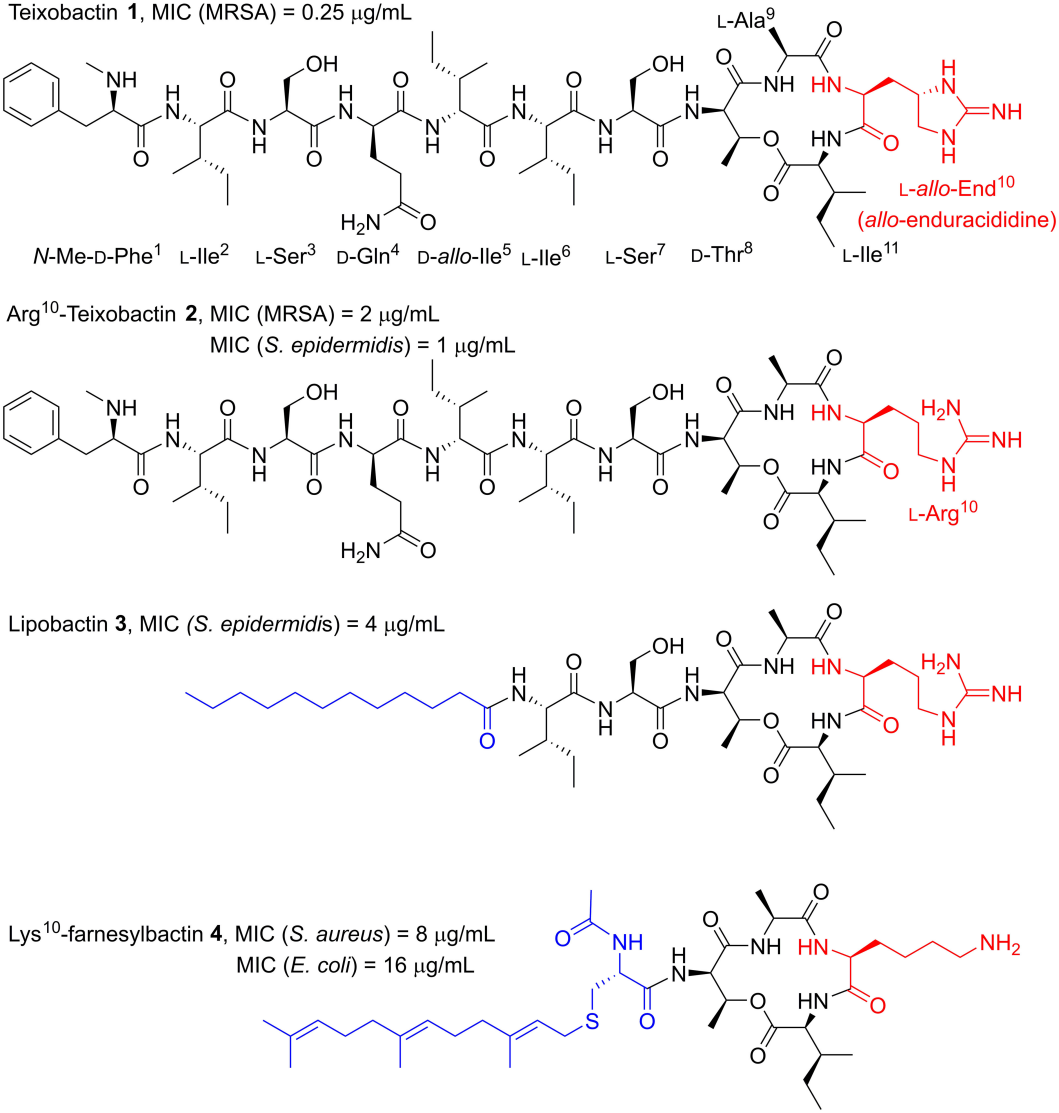
Structures of teixobactin **1** and teixobactin analogs; Arg^10^-teixobactin **2**, lipobactin **3**, and Lys^10^-farnesylbactin **4**. Examples of most relevant MICs from the literature are shown above each structure. Yang H. et al. (2016), Girt et al. (2018), Zong et al. (2018) The End^10^, Arg^10^ and Lys^10^ substitutions (red) and the lipid moieties of lipobactin **3** and farnesylbactin **4** (blue) are highlighted.

Nature has historically been the primary source of medicinally important antibiotics (Moloney, 2016). By screening soil microorganisms, Ling et al. discovered the novel antimicrobial peptide (AMP) teixobactin (**1**, Figure 1), isolated from bacterium *Eleftheria terrae* (Ling et al., 2015). Teixobactin exhibited potent activity against Gram-positive pathogens with antimicrobial resistance (AMR) such as methicillin-resistant *Staphylococcus aureus* (MRSA [MIC = 0.25 μg/ml]). Crucially, resistance in *S. aureus* was not induced by exposure to sub-lethal doses of teixobactin. The lack of resistance was found to be due to teixobactin binding to the highly conserved targets lipid II and lipid III. As both of these are non-protein precursors to the bacterial cell wall, they cannot be easily mutated to impart AMR.

Structurally, teixobactin is an undecapeptide with a cyclic tetradepsipeptide and a seven-residue exocyclic chain comprised of four d-amino acid residues and a rare l-*allo*-enduracididine (End) residue (Atkinson et al., 2016). The total synthesis of teixobactin has been independently reported by the Li and Payne groups, as well as the complete solution-phase total synthesis by the Reddy group (Giltrap et al., 2016; Jin et al., 2016; Gunjal and Reddy, 2019). Collectively, it was established that the End residue, containing a cyclic guanidine moiety, was the bottleneck for developing an efficient synthesis of teixobactin, limiting its potential as a drug candidate. This is due to the preparation of the End building block requiring a lengthy and low-yielding synthetic route.

The Albericio group (Jad et al., 2015) demonstrated that End can be substituted for arginine (l-Arg^10^-teixobactin **2**, Figure 1), leading to a much simpler and overall higher-yielding synthetic route. Despite this substitution, the l-Arg^10^-teixobactin analog maintained excellent, albeit reduced potency, with MIC values ranging 1–4 μg/mL across a number of Gram-positive bacterial species (Yang H. et al., 2016; Zong et al., 2018). The rationale for this substitution being that the positive charge from the guanidinium group was the moiety responsible for eliciting antibacterial activity. Indeed, other positively charged isosteres have been substituted that have exhibited antimicrobial activity. These include lysine, ornithine, histidine, and homoarginine, to name a few (Schumacher et al., 2017; Matheson et al., 2019).

In light of the fact that End is not essential for antimicrobial activity, many ensuing reports of synthetic analogs used l-Arg^10^-teixobactin as the starting point to conduct structure-activity relationship (SAR) studies of teixobactin (Abdel Monaim et al., 2016, 2017, 2018; Parmar et al., 2016, 2017a,b,c, 2018; Yang H. et al., 2016; Chen et al., 2017; Jin et al., 2017, 2018; Schumacher et al., 2017; Wu et al., 2017; Yang et al., 2017; Girt et al., 2018; Zong et al., 2018) Employing this strategy, the Nowick group (Yang H. et al., 2016) demonstrated that the teixobactin macrocycle was an important pharmacophore, as a linear Arg^10^ analog was inactive. In the same work, they examined the role of the *N*-terminal tail by replacing residues 1–5 with a 12-carbon linear alkyl lipid. The resultant analog retained potent antimicrobial activity (MIC 4–8 μg/mL), albeit with an MIC two- or four-fold higher than l-Arg^10^-teixobactin (MIC 1–4 μg/mL) across a range of Gram-positive bacterial species. This lipophilic analog was coined “lipobactin” **3** (Figure 1). It is understood that the *N*-terminal tail interacts with the plasma membrane to deliver teixobactin into the vicinity of the binding targets, lipid II, and lipid III, in order to confer antibacterial activity (Yang H. et al., 2016). Lipobactin later inspired the work of the Jamieson group (Girt et al., 2018), who undertook the synthesis of farnesyl- and geranyl-derived lipopeptidomimetics of teixobactin with residues 1–7 truncated. The most potent of the analogs, Lys^10^-farnesylbactin **4** (Figure 1), elicited activity against both Gram-positive and Gram-negative bacteria with moderate potency.

**Scheme 1:**
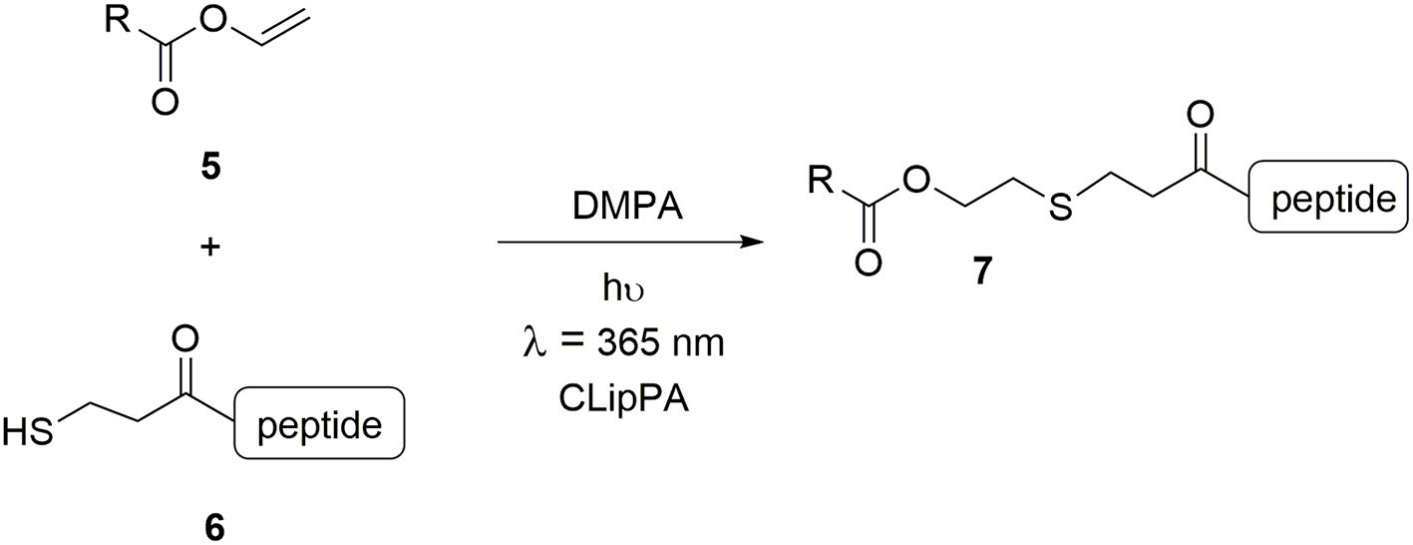
Vinyl ester **5** and peptide **6** with a free thiol is irradiated under UV at 365 nm with a photoinitiator (DMPA) to generate *S*-lipidated peptide **7**.

The work reported herein showcases a technique developed in our group for the facile synthesis of an array of lipopeptidomimetics: a modified **C**ysteine **Lip**idation on a **P**eptide and **A**mino acid (CLipPA), whereby cysteine was substituted for 3-mercaptopropionic acid (3-MPA) (Scheme 1) (Wright et al., 2013; Yang S. et al., 2016; Brimble et al., 2017; Hermant et al., 2020; Yang et al., 2020). CLipPA comprises a one-pot thiol-ene reaction between a vinyl ester bearing a lipid **5** and a peptide containing a free thiol handle **6** in which irradiation at 365 nm in the presence of photoinitiator 2,2-dimethoxy-2-phenylacetophenone (DMPA) forms a thioether-linked lipopeptide **7**.

**Figure 2:**
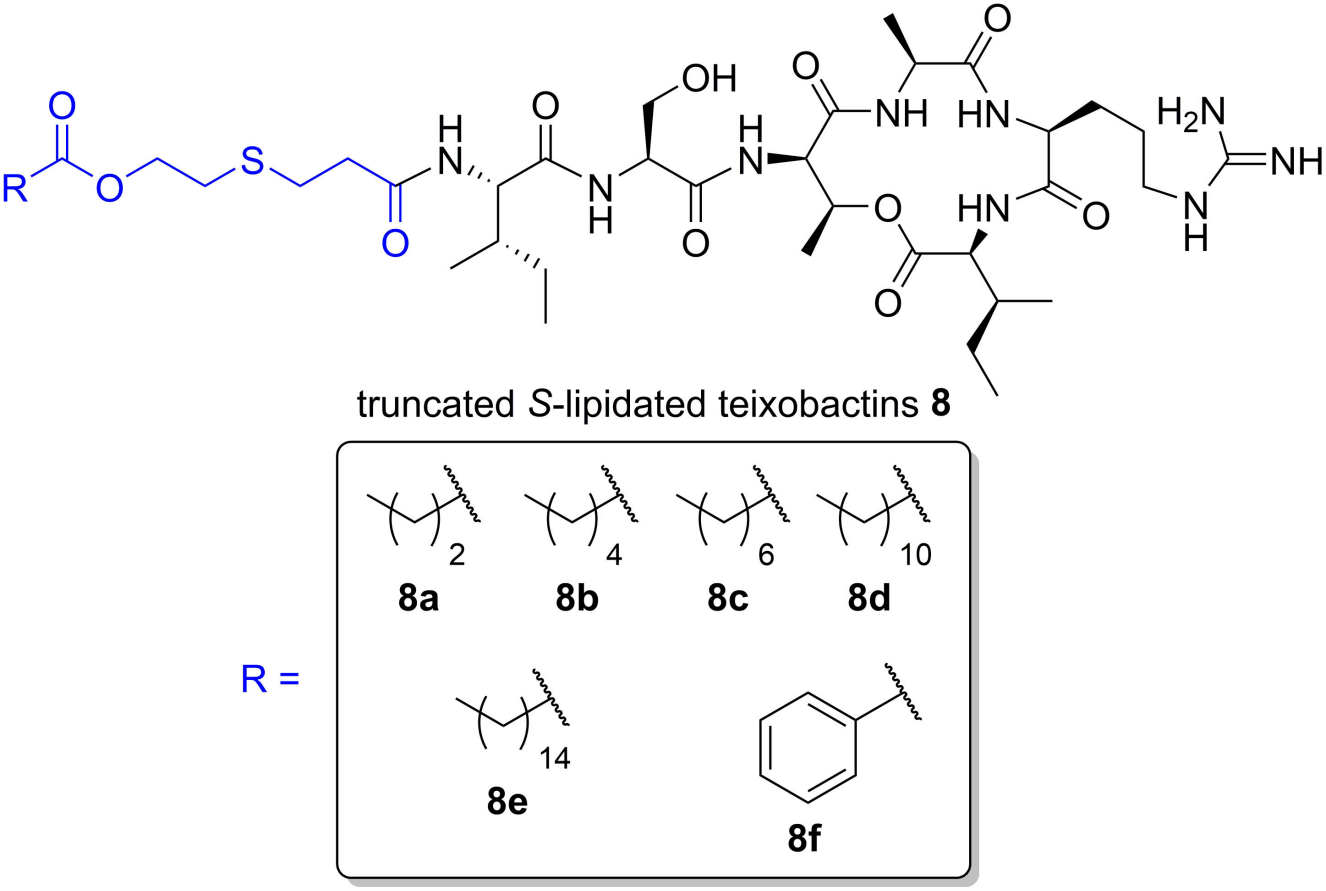
General structure of truncated *S*-lipidated teixobactin analog **8** with the *S*-lipidated moiety (blue). The *R* group denotes the truncated *S*-lipidated teixobactin derivatives **8a**–**8f** (boxed) prepared in the current work.

We have recently prepared a series of *S-*lipidated analogs of the cyclic lipopeptide iturin A by employing CLipPA thiol-ene chemistry (Yim et al., 2020). Taking a similar approach, we envisaged the dodecanoyl chain from lipobactin **3** could be replaced with *S*-lipidated derivatives of 3-mercaptopropionic acid (MPA) to afford truncated *S*-lipidated teixobactin analogs **8** (Figure 2). MPA is a structural analog of cysteine lacking the amine group. Compared to MPA, use of cysteine results in an extra charge by protonation of its *N*^α^-amino group at physiological pH and would thereby render the analogs significantly different to the parent compound. Hence, MPA (lacking an additional amino group) was selected as the thiol handle for the generation of *S*-lipidated teixobactin analogs.

## Results and Discussion

**Scheme 2:**
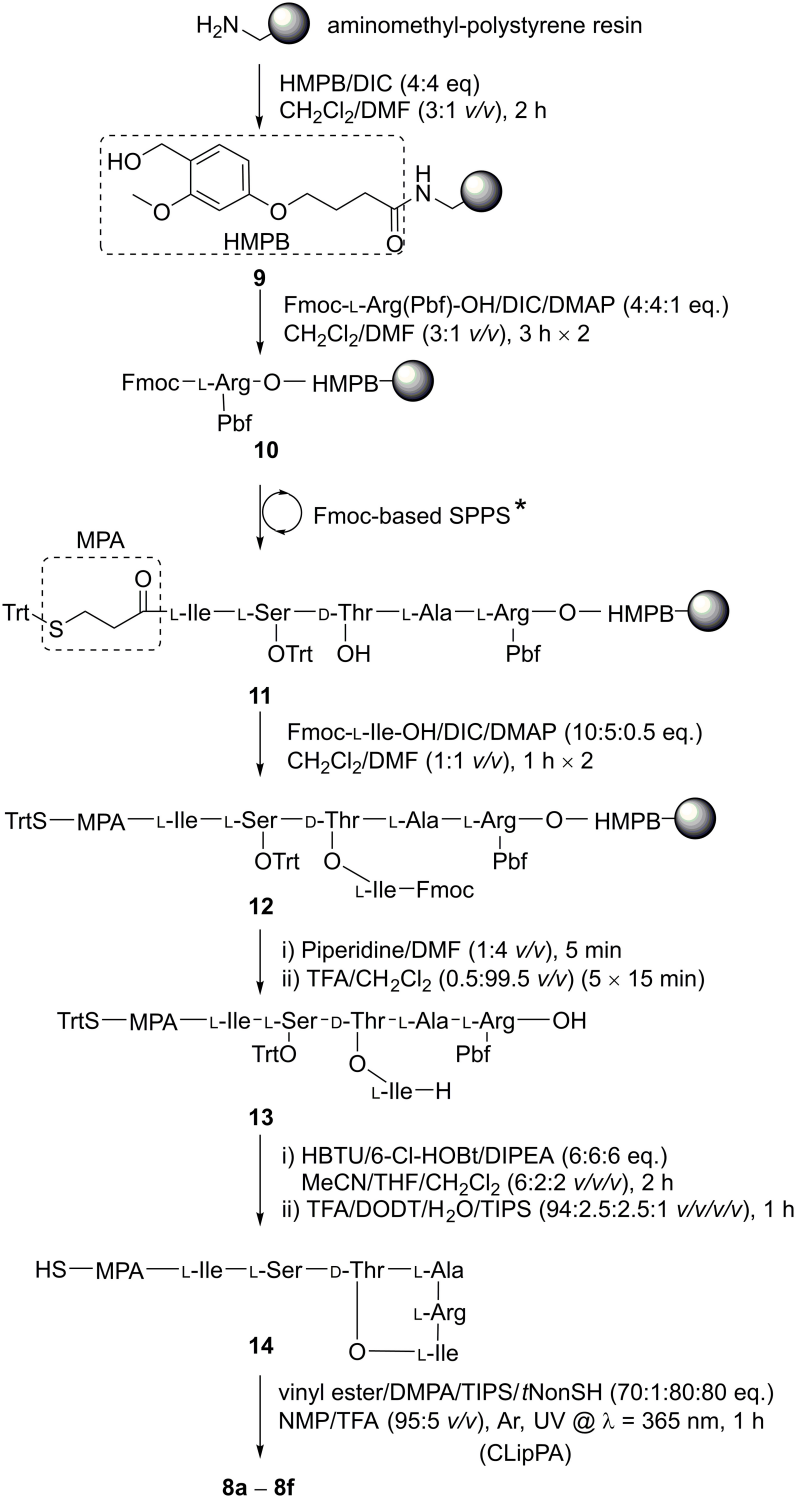
Synthesis of **8a−8f**. The linear sequence was assembled using Fmoc-SPPS under microwave irradiation: (i) Fmoc-deprotection: piperidine/DMF (1:4 *v/v*), 50 W, 75°C, 2 × 3 min; (ii) coupling of amino acids: Fmoc-AA-OH (4 eq.), HATU (3.8 eq.), DIPEA (8 eq.), 25 W, 50°C, 5 min in DMF. *d-Thr-OH was coupled without side chain protection.

The three key steps for the synthesis of **8** are ester-bond formation, macrolactamization, and finally attachment of the lipid moiety (Scheme 2).

We based our synthesis on that described by the Nowick group (Yang H. et al., 2016), who performed the key cyclization between Arg and Ile in solution. In order to prepare a fully protected peptide suitable for cyclization in solution, a linker that is sensitive to weakly acid conditions was required. (4-Hydroxymethyl-3-methoxyphenoxy)butyric acid (HMPB) linker allows peptide detachment from the resin using 0.5 % trifluoroacetic acid (TFA) (McMurray and Lewis, 1993; Góngora-Benítez et al., 2013), thereby allowing for a precursor to be liberated from the resin with side chain protecting groups intact and the *C*-terminus free for lactamization with the *N*^α^-amine of the branched Ile residue. HMPB was coupled onto aminomethyl-polystyrene resin using *N,N'*-diisopropylcarbodiimide (DIC) to give linker-resin **9**. Following the method reported by the Nowick group (Yang H. et al., 2016), **10** was formed by attaching Fmoc-l-Arg(Pbf)-OH to HMPB using DIC with catalysis by 4-dimethylaminopyridine (DMAP). Through iterative deprotections using 20% piperidine in DMF (*v/v*) and subsequent coupling steps using commercially-available amino acid building blocks activated by 2-(7-aza-1*H*-benzotriazole-1-yl)-1,1,3,3-tetramethyluronium hexafluorophosphate (HATU) and *N,N*-diisopropylethylamine (DIPEA) under microwave irradiation, resin **10** was elongated to afford peptidyl resin **11**. A ninhydrin test showed that all couplings were complete (Sarin et al., 1981). Akin to the Nowick group synthesis (Yang H. et al., 2016), d-Thr without side chain protection could be used to facilitate the subsequent side chain esterification step as we observed no *O*-acylation during sequence elongation. Initially, for Ser (Jad et al., 2015), we employed *tert*-butyl (*t*Bu) side chain protection. Upon final global deprotection, LC-MS analysis of **14** revealed an *S*-*t*Bu adduct of the MPA group **SI 1** (*ca*. 33%, Figure S1) that could not be reversed. Hence, we switched to trityl (Trt) protection for the hydroxyl group of serine, alleviating the problem.

Resin bound peptide **11** was then *O*-acylated on d-Thr with two treatments of Fmoc-Ile-OH (10 eq.) activated with DIC (5 eq.) and DMAP (0.5 eq.), giving branched depsipeptidyl resin **12**. LC-MS analysis showed the esterification was efficient with a conversion of *ca*. 93% after two coupling cycles. A final *N*^α^-Fmoc deprotection was achieved with one treatment of 20% piperidine/DMF (*v/v*). The branched depsipeptide was then released from the solid support with repeated 15-min treatments of TFA/CH_2_Cl_2_ (0.5:99.5 *v/v*) until a pink colouration on the resin was observed, (*ca*. 5 iterations) to afford the side chain protected cyclization precursor **13** in 37% crude yield.

Cyclization of crude **13** was effected using HBTU/6-Cl-HOBt/DIPEA (6:6:6 eq.) in a mixture of MeCN/THF/CH_2_Cl_2_ (6:2:2 *v/v*/*v*) for 2 h. The solvent mixture could be readily evaporated, conveniently permitting the subsequent addition of the cleavage cocktail to remove side chain protecting groups directly. HPLC purification of the fully unprotected cyclic depsipeptide afforded pure **14**, ready for *S*-lipidation, in 51% crude yield and 80% purity. Depsipeptide **14** was dissolved in NMP/TFA (95:5 *v/v*) along with radical initiator DMPA, vinyl ester and the scavengers, TIPS and *t*NonylSH (1:70:80:80 eq. based on **14**) under argon (Yang et al., 2020). The cocktail was irradiated with a UV lamp at 365 nm and stirred for 1 h at room temperature. Employing different vinyl esters, we were able to construct a series of seven truncated *S*-lipidated teixobactin analogs **8a**–**8f** (Figure 2) from the common precursor **14**, yielding 10–13% after purification.

We evaluated the antibiotic activity of the analogs **8a**–**8f** against *S. aureus* using lipobactin **3** as a reference compound. Lipobactin **3** was prepared using Fmoc-SPPS as described by the Nowick group (Yang H. et al., 2016) and purified to 95% (Yang H. et al., 2016). Lipobactin **3** demonstrated an MIC of 8 μM against *S. aureus* ATCC 29213 (*ca*. 6.5 μg/mL, Table S1), which was within two-fold of the reported MIC against *S. epidermidis* ATCC 14990 (4 μg/mL) (Yang H. et al., 2016). Despite the distinct similarity to lipobactin **3**, in which hydrophobic residues 1–5 of teixobactin are also replaced with a lipid moiety, the *S*-lipidated teixobactin analogs **8a**–**8f** failed to demonstrate antibacterial activity against *S. aureus* (MIC > 128 μM, Table S1). To examine the reasons for the lack of bioactivity, the hydrophobicity of the *S*-lipidated teixobactin analogs as a function of lipid tail length were examined by analyses of their RP-HPLC retention times and compared to that of lipobactin **3** (Table 1).

**Table 1:** RP-HPLC column retention times (*t*_*R*_) of the synthesized compounds and the comparison to lipobactin **3** as a function of lipid tail length.

**Compound**	***t_***R***_* (min)**	**Δt (min)**	**Δ atoms**
**3**	20.38	0	0
**8a**	14.98	−5.4	−1
**8b**	16.88	−3.5	+1
**8f**	15.93	−4.5	+2
**8c**	18.89	−1.5	+3
**8d**	23.21	+2.8	+7
**8e**	27.98	+7.6	+11

Analogs **8a** and **8b** are most similar in atom length with respect to lipobactin **3**, with a difference of one carbon shorter and longer, respectively. Both analogs are however, more hydrophilic than **3** as judged by their earlier retention times. Based on this observation, we suspect that the bridging unit, –(O)COCH_2_CH_2_SCH_2_CH–, imparts undesirable hydrophilicity onto the lipid tail as judged by RP-HPLC, likely arising from the additional ester moiety and potentially the thioether moiety as well. It was observed that *S*-lipidated teixobactin compounds which are more hydrophobic than **3** require an atom difference of +7 or more (**8d** and **8e**), thereby significantly increasing the length of the tail portion. We therefore suggest that the presence of this bridging unit may interfere with *S*-lipidated teixobactin compounds traversing into the bacterial plasma membrane or with the interactions toward lipids II and III, thereby destroying any antibiotic activity against *S. aureus* as observed in our MIC assays.

## Conclusions

Despite the lack of antibiotic activity, lipidated teixobactin analogs were successfully synthesized using an efficient process that was achieved using commercially available vinyl ester building blocks. The advantage of this technique over classical lipidation is the ability to chemoselectively introduce a diverse range of functionalities onto the unprotected cyclic teixobactin pharmacophore. Moreover, the synthetic route leading to the thiolated precursor (the teixobactin pharmacophore bearing a thiol handle) was well-established, facilitating the subsequent one-step modified CLipPA reaction.

The disappointing lack of antimicrobial activity exhibited by the truncated *S*-lipidated teixobactin analogs warrants further investigation into the effect of the thioether bridging unit upon the key interactions with the intended bacterial membrane targets. Modeling the interactions of the truncated *S*-lipidated teixobactin analogs with the bacterial membrane, comparing the thioether-linked lipid to both a simple alkyl chain (the lipid tail of lipobactin), and the *N*-terminal residues of teixobactin, would likely provide interesting mechanistic insights for this unique class of AMP.

## Data Availability Statement

The raw data supporting the conclusions of this article will be made available by the authors, without undue reservation, to any qualified researcher.

## Author Contributions

All synthetic work and data analysis were carried out by VY. Antibacterial susceptibility testing was carried out by VY under supervision of AC. The experiment design and manuscript preparation were done by VY, AC, IK, PH, and MB. All experimental work was carried out in the laboratory of MB. All authors have approved the submitted manuscript.

## Conflict of Interest

The authors declare that the research was conducted in the absence of any commercial or financial relationships that could be construed as a potential conflict of interest.

## Supplementary Material

Procedures for the synthesis of lipobactin and CLipPAbactin analogs and minimum inhibitory concentration (MIC) assays.
