# Supplementary material for: Thiol-ene Enabled Chemical Synthesis of Truncated S-Lipidated Teixobactin Analogs
Source: Front Chem. 2020 Aug 4;8:568. doi: 10.3389/fchem.2020.00568 (PMC7417771; doi:10.3389/fchem.2020.00568)
Supplement: Supplementary file 1 [file Data_Sheet_1.docx]

***Supplementary Information***

Thiol-ene Enabled Chemical Synthesis of Truncated *S*-Lipidated Teixobactin Analogs

**Victor V. Yim,^1,2^ Alan J. Cameron,^1,2^ Iman Kavianinia,^1,2,3^ Paul W. R. Harris,^1,2,3^* and Margaret A. Brimble^1,2,3^***

^1^School of Biological Sciences, University of Auckland, 3A Symonds St, Auckland 1010, New Zealand

^2^School of Chemical Sciences, University of Auckland, 23 Symonds Street, Auckland 1010, New Zealand.

^3^Maurice Wilkins Centre for Molecular Biodiscovery, University of Auckland, 3A Symonds Street, Auckland 1010, New Zealand.

*** Correspondence:**Associate Professor Paul W. R. Harris

paul.harris@auckland.ac.nz

Distinguished Professor Margaret A. Brimble
m.brimble@auckland.ac.nz

**Table of Contents**

[S1. Abbreviations 2](#_Toc33654679)

[S2. General Information 2](#_Toc33654680)

[S3. General Methods 3](#_Toc33654681)

[S4. Experimental section 7](#_Toc33654682)

[S4.1. Synthesis of truncated *S*-lipidated teixobactin analogs **8a** – **8f** 7](#_Toc33654683)

[S4.1.1. Synthesis of linear peptidyl resin **11**. 7](#_Toc33654684)

[S4.1.2. Esterification to generate branched depsipeptidyl resin **12**. 10](#_Toc33654685)

[S4.1.3. Resin cleavage to generate branched depsipeptide **13**. 11](#_Toc33654686)

[S4.1.4. Lactamization and deprotection to cyclic depsipeptide **14**. 13](#_Toc33654687)

[S4.1.5. Synthesis of truncated *S*-lipidated teixobactin analog **8a**. 14](#_Toc33654688)

[S4.1.6. Synthesis of truncated *S*-lipidated teixobactin analog **8b**. 15](#_Toc33654689)

[S4.1.7. Synthesis of truncated *S*-lipidated teixobactin analog **8c**. 16](#_Toc33654690)

[S4.1.8. Synthesis of truncated *S*-lipidated teixobactin analog **8d**. 17](#_Toc33654691)

[S4.1.9. Synthesis of truncated *S*-lipidated teixobactin analog **8e**. 18](#_Toc33654692)

[S4.1.10. Synthesis of truncated *S*-lipidated teixobactin analog **8f**. 19](#_Toc33654693)

[S4.2. Synthesis of lipobactin **3** 20](#_Toc33654694)

[S5. Antibacterial susceptibility testing 22](#_Toc33654695)

[S6. References. 22](#_Toc33654696)

# Abbreviations

°C: degrees celcius. 6-Cl-HOBt: 6-chloro-1-hydroxybenzotriazole. A = ampere (s). Ar: argon. *ca.*: *circa*/approximately. CLipPA: Cysteine Lipidation on a Peptide or Amino acid. DIC: *N,N′*-diisopropylcarbodiimide. DIPEA: *N,N*-diisopropylethylamine. DMF: *N,N*-dimethylformamide. DMPA: 2,2-dimethoxy-2-phenylacetophenone. DODT: 2,2’-(ethylenedioxy)-diethanethiol. eq.: molar equivalence. ESI-MS: electrospray ionization. Fmoc: (9*H*-fluoren-9-yl)methoxycarbonyl. h: hour(s). HATU: *O*-(7-azabenzo-triazol-1-yl)-1,1,3,3*-*tetramethyluronium hexafluorophosphate. HBTU: *O*-(benzotriazol-1-yl)-1,1,3,3-tetramethyluronium hexafluorophosphate. HMPB: (4-hydroxymethyl-3-methoxyphenoxy)butyric acid. HPLC: high performance liquid chromatography. Hz: Hertz. hν: light. LC-MS: liquid chromatography-mass spectrometry. Me: methyl. MeCN: acetonitrile. MeOH: methanol. mg: milligram. min: minute (s). mL: millilitres. mmol: millimoles. *m/z*: mass per charge. MH: Mueller Hinton. nm: nanometer. NMP: *N*-methyl-2-pyrrolidone. Pbf: 2,2,4,5,7-pentamethyldihydrobenzofuran-5-sulfonyl. r.t.: room temperature. RP: reverse phase. *R_t_*: retention time. Ser: serine. SPPS: solid-phase peptide synthesis. *t*Bu: *tert*-butyl. *t*Non: *tert*-nonyl. TFA: trifluoroacetic acid. TIPS: triisopropylsilane. *t*NonSH: *tert*-nonyl mercaptan. Trt: triphenylmethyl. UV: ultraviolet. V: volts. *v/v*: volume per volume. W: Watt (s). w.r.t.: with respect to. λ: lambda/wavelength. μL: microliter. μm: micrometre.

# General Information

All reagents were purchased as reagent grade and used without further purification unless otherwise noted. THF was purchased from Avantor (Radnor, PA). Argon was purchased from BOC group (Guildford, UK). CH_2_Cl_2_, MeOH and diethyl ether were purchased from ECP (Auckland, New Zealand). MeCN was purchased from Fisher Scientific (Hampton, NH). Fmoc-Arg(Pbf)-OH, Fmoc-Ala-OH, Fmoc-Ser(*t*Bu)-OH, Fmoc-Ile-OH, HATU and HBTU were purchased from GL Biochem (Shanghai, China). Fmoc-Ser(Trt)-OH was purchased from Peptides International (Louisville, KY). Fmoc-d-Thr-OH and 6-Cl-HOBt were purchased from AAPPTec (Louisville, KY). HMPB linker and DMAP was purchased from Merck (Darmstadt, Germany). TFA was purchased from Oakwood Chemicals (Estill, NC). DMF was purchased from Scharlau (Barcelona, Spain). DIC, DIPEA, DMPA, DODT, lauric acid, 3-(tritylthio)propionic acid, NMP, piperidine, TIPS, *t*NonSH, vinyl benzoate and vinyl butyrate were purchased from Sigma-Aldrich (St. Louis, MO). Aminomethyl-polystyrene resin was purchased from Rapp polymere (Tübingen, Germany). Vinyl laurate, vinyl hexanoate, vinyl octanoate and vinyl palmitate were purchased from TCI (Tokyo, Japan). H_2_O was purified using a Sartorius (Göttingen, Germany) arium® pro ultrapure water system.

Microwave irradiated reactions were performed in a CEM (Matthews, NC) Discover SP, Model 908010 reactor. Dibenzofulvene measurements for the Fmoc release assay was performed in a Shimadzu (Kyoto, Japan) UV-1280 spectrophotometer at 290 nm. Analytical RP-HPLC was used to analyse final compounds and performed on a Dionex (Sunnyvale, CA) UltiMate 3000 system using a Waters (Milford, MA) Xterra MS C18 (5 μm 4.6 × 150 mm) column, and Chromeleon software was used for data processing. Buffer A: 0.1% (*v/v*) TFA in H_2_O; buffer B: 0.1% (*v/v*) TFA in MeCN. LC-MS was performed on an Agilent (Santa Clara, CA) 1260 Infinity with UV absorbance at λ = 214 nm equipped with an Agilent 6120 Quadrupole LC-MS using an Agilent Zorbax 300SB-C3 column (3.5 μm, 3.0 × 150 mm) column with a linear gradient of 5 – 95% B (*ca.* 3% B/min) at r.t., with a flow rate of 0.3 mL/min. Data processing was carried out by Agilent OpenLAB software. Buffer A: 0.1% (*v/v*) formic acid in H_2_O; buffer B: 0.1% (*v/v*) formic acid in MeCN. Crude peptides were purified on Dionex UltiMate 3000 preparative HPLC using Agilent Zorbax 300SB-C18 column (5 μm, 9.4 × 250 mm) and Chromeleon software was used for data processing. Ultraviolet irradiation was carried out using Spectroline (Westbury, NY) hand-held lamp EA-160/FA, 6W integrally filtered tube at 50 Hz, 0.17 A and λ = 365 nm. Biological assays were carried out inside a Thermo Fisher (Waltham, MA) HERAsafe KSP 12 biological safety cabinet on 96-well plates obtained from MediRay (Auckland, New Zealand). Mueller Hinton (MH) broth and *Staphylococcus aureus* ATCC 29213 was obtained from Thermo Fisher. Media and bacteria were incubated in N-Biotek (Geyonggi, South Korea) NB205L shaker.

# General Methods

***General Method A: loading the linker***

To aminomethyl polystyrene resin (397 mg, 0.5 mmol, 1.26 mmol/g) pre-swollen in CH_2_Cl_2_/DMF (1:1 *v/v*, 5 mL) was added HMPB linker (480 mg, 2.0 mmol, 4 eq.) and DIC (313 μL, 2.0 mmol, 4 eq.) dissolved in CH_2_Cl_2_/DMF (1:4 *v/v*, 5 mL) and gently agitated for 2 h at r.t to afford resin **9**. The completion of the coupling was monitored using the ninhydrin test.^1^ If the coupling was incomplete, the coupling procedure was repeated with freshly prepared reagents.

***General Method B: loading the first residue***

Fmoc-Arg(Pbf)-OH (1.3 g, 2.0 mmol, 4 eq.) and DIC (313 μL, 2.0 mmol, 4 eq.) was dissolved in CH_2_Cl_2_/DMF (3:1 *v/v*, 10 mL) at 0 °C and stirred for 30 minutes and the solution was added to resin **9** (0.5 mmol, 1 eq.). DMAP (61 mg, 0.5 mmol, 1 eq.) pre-dissolved in DMF (200 μL) was added to the resin mixture and gently agitated for 3 h and repeated with fresh reagents. The solution was drained and washed with DMF (3 × 5 mL) and the resin was dried by washing with CH_2_Cl_2_ (3 × 5 mL) to afford resin **10**. A small portion of the resin (*ca.* 1 mg) was mixed in piperidine/DMF (1:4 *v/v*, 5 mL) and the absorbance of dibenzofulvene was measured with a UV spectrophotometer at 290 nm to estimate the esterification with **Equation S1** and **Equation S2**.^2^ The loading was estimated to be 51% (0.26 mmol, 0.64 mmol/g), which was and then used to define equivalents in all subsequent steps.

**Equation S1.** Fmoc determination.

$$A_{t}= \frac{B\times1000}{1000+\left[ B\left( M-X \right) \right]}$$

$A_{t}$ = theoretical substitution (mmol/g)
$B$ = substitution of starting resin (mmol/g)
$M$ = molecular weight of target peptide, plus all protecting group
$X$ = 17 for aminomethyl resin

**Equation S2.** Determining actual substitution ($A$)

$A=\frac{{Abs}_{sample}}{m_{sample}\times1.75}$; $\% loading= \frac{A}{A_{t}}$

$A$ = substitution (mmol/g)
$m_{sample}$ = mass of sample in mg

***General Method C: elongation of the sequence***

*N*^α^-Fmoc-removal from **10** was performed with piperidine/DMF (1:4 *v/v*) under microwave irradiation (50 W, 75 °C, 2 × 3 min), the solution was then drained, and the resin was washed with DMF (3 × 5 mL). *N^α^*-protected amino acids (4 eq.) were coupled onto the free amino group in DMF with HATU (3.8 eq.) and DIPEA (8 eq.) under microwave irradiation (25 W, 50 °C, 5 min), the solution was then drained and the resin was washed with DMF (3 × 5 mL). If a ninhydrin test was required, the resin was dried by washing with MeOH (3 × 5 mL). Deprotection and coupling steps were repeated for each amino acid until the completed linear resin-bound peptide sequence (**11** or **15**) was afforded. To confirm the identity of the assembled linear sequence, the peptide was cleaved by treating a small fraction of the resin to a cleavage cocktail and analysed by LC-MS according to **General Method D.**

***General Method D: acid cleavage of the peptide off the resin and LC-MS***

The peptide was released from the solid support by treatment with TFA/DODT/H_2_O/TIPS (94:2.5:2.5:1 *v/v/v/v*) for 2 h. The solution was filtered, and the filtrate was sparged with nitrogen to evaporate TFA. The residue was triturated with diethyl ether (4 °C), centrifuged and the supernatant decanted to give the crude peptide as a pellet. The pellet was dissolved in H_2_O:MeCN (1:1 *v/v*) containing 0.1% TFA and a small aliquot was taken for LC-MS analysis. The remaining solution was lyophilized and analysed by LC-MS with an Agilent Zorbax 300SB-C3 (3.5 μm, 3.0 × 150 mm) column using a linear gradient of 5 – 95% B (*ca.* 3% B/min) at r.t., with a flow rate of 0.3 mL/min.

***General Method E: generating side-chain protected peptide***

The appropriate branched depsipeptidyl resin was subjected to TFA/CH_2_Cl_2_ (0.5:99.5, *v/v*, 5 mL) for 15 mins. The solution was drained and the filtrate was sparged with nitrogen to evaporate TFA. This process was repeated (*ca.* 5 iterations) until the resin takes on a pink coloration and the residues combined. The residue was dried under reduced pressure, dissolved in H_2_O:MeCN (1:1 *v/v*) containing 0.1% TFA and lyophilized to give the desired branched side chain protected depsipeptide.

***General Method F: macrocyclization and deprotection***

1. The desired branched side chain protected peptide (1 eq.), HBTU (6 eq.) and 6-Cl-HOBt (6 eq.) were dissolved in a mixture of MeCN/THF/CH_2_Cl_2_ (6:2:2 *v/v/v*) with a final peptide concentration of 0.5 mg/mL. The mixture was stirred for 30 min at r.t. and DIPEA (6 eq.) was added slowly and further stirred for another 2 h at r.t at which time the solvent was removed under reduced pressure.
2. The side chain protecting groups were removed with a cleavage cocktail of TFA/DODT/H_2_O/TIPS (94:2.5:2.5:1 *v/v*/*v/v*, 10 mL) for 1 h. The solution was sparged with nitrogen to evaporate TFA, triturated with diethyl, centrifuged and the supernatant decanted to give the crude peptide as a pellet. The pellet was dissolved in H_2_O:MeCN (1:1 *v/v*) containing 0.1% TFA and lyophilized to give the crude cyclic peptide.

***General Method G: direct conjugation of vinyl esters and free thiol-containing peptide using modified CLipPA***

Cyclic peptide **14** (1 eq.), DMPA (1 eq.), vinyl ester (70 eq.), and TIPS (80 eq.) were dissolved in NMP with a peptide final concentration of 10 mg/mL and sparged with argon for 15 min. *t*NonSH (80 eq.) and TFA (5% *v/v* w.r.t. NMP) were then added under argon and the mixture stirred with irradiation at 365 nm for *ca.* 1 h at r.t and progress monitored by LC-MS. Upon completion of the reaction, the mixture was triturated with diethyl ether, centrifuged and the supernatant decanted to give the crude peptide as a pellet. The pellet was dissolved in H_2_O:MeCN (1:1 *v/v*) containing 0.1% TFA and lyophilized. The crude mixture was then purified according to **General Method H** to afford **8a** – **8f**.

***General Method H: purification***

Crude peptide was dissolved in 0.1% (*v/v*) TFA in H_2_O:MeCN (4:1 *v/v*), centrifuged and filtered using Phenomenex (Torrance, CA) Phenex syringe filters (26 mm, 0.45 μm). The filtrate was injected as 2500 μL aliquots and purified using a slow gradient on RP-HPLC (1 – 95% B, 1% B/min, 4 mL/min).^3^ The fractions were collected based on UV absorbance at wavelengths of 210 nm, 230 nm, 254 nm and 280 nm, followed by mass-spectrometry analysis (ESI^+^ 100 V; H_2_O:MeCN; 1:1 *v/v*, 0.2 mL/min). The fractions containing the purified peptide were combined and lyophilized.

# Experimental section

## Synthesis of truncated *S-*lipidated teixobactin analogs 8a – 8f

### Synthesis of linear peptidyl resin **11**.

Ester resin **10** (0.26 mmol, 0.64 mmol/g) was elongated with amino acids (1.02 mmol, 4 eq.) and HATU (368 mg, 0.97 mmol, 3.8 eq.) DIPEA (342 μL, 2.0 mmol, 8 eq.) according to **General Method C** in the order of Fmoc-Ala-OH (318 mg), Fmoc-d-Thr-OH (348 mg), Fmoc-Ser(*t*Bu)-OH (391 mg), Fmoc-Ile-OH (360 mg) and 3-(tritylthio)propionic acid acid (355 mg) to generate peptidyl resin **11**. Analysis by LC-MS according to **General Method D** revealed desired peptide **SI 1** (67%, by peak area); LC-MS: *t_R_* = 9.7 min, [M + H]^+^ found 635.3, [C_25_H_46_N_8_O_9_S + H]^+^ requires 635.3, and an *S*-*t*Bu adduct **SI 2** during resin cleavage and global deprotection (33%, by peak area); LC-MS: *t_R_ =* 10.5 min, [M + H]^+^ found 691.4, [C_29_H_54_N_8_O_9_S + H]^+^ requires 691.4. Re-synthesis by substituting Fmoc-Ser(*t*Bu)-OH for Fmoc-Ser(Trt)-OH (581 mg) in Fmoc-SPPS according to **General Method C** eliminated **SI 2** (**Figure S1**).


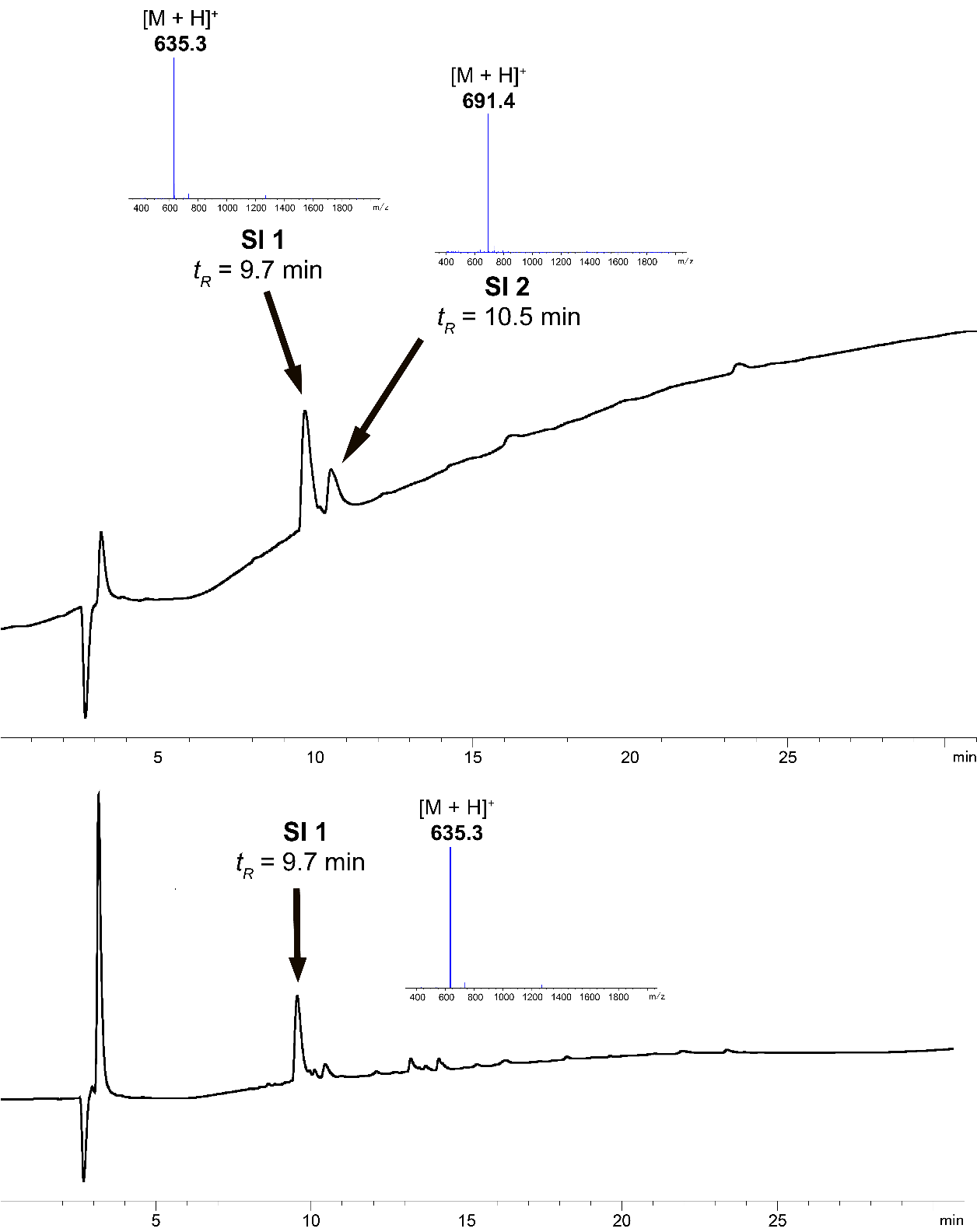


**Figure S1.** LC-MS analyses at 214 nm of peptidyl resin **11**, represented by cleavage product **SI 1**. **SI 2** depicts the cleavage *S*-*t*Bu by-product. Top chromatogram refers to peptide synthesized with Fmoc-Ser(*t*Bu)-OH, bottom chromatogram refers to peptide synthesized with Fmoc-Ser(Trt)-OH.

### Esterification to generate branched depsipeptidyl resin **12**.

Following **General Method B,** with the following modifications, Fmoc-Ile-OH (901 mg, 2.6 mmol, 10 eq.) was esterified onto peptidyl resin **11** with DIC (200 μL, 1.3 mmol, 5 eq.) and DMAP (16 mg, 0.13 mmol, 0.5 eq.). The conversion rate was measured by relative LC-MS peak area of the starting material and product after cleaving a small portion of the resin according to **General Method D**. LC-MS (5 – 95% B, 3% B/min, 0.3 mL/min) estimated the esterification to the branched depsipeptide **SI 3** to be 93%; LC-MS: *t_R_ =* 19.4 min, [M + H]^+^ found 970.5, [C_29_H_54_N_8_O_9_S + H]^+^ requires 970.5 (**Figure S2**).


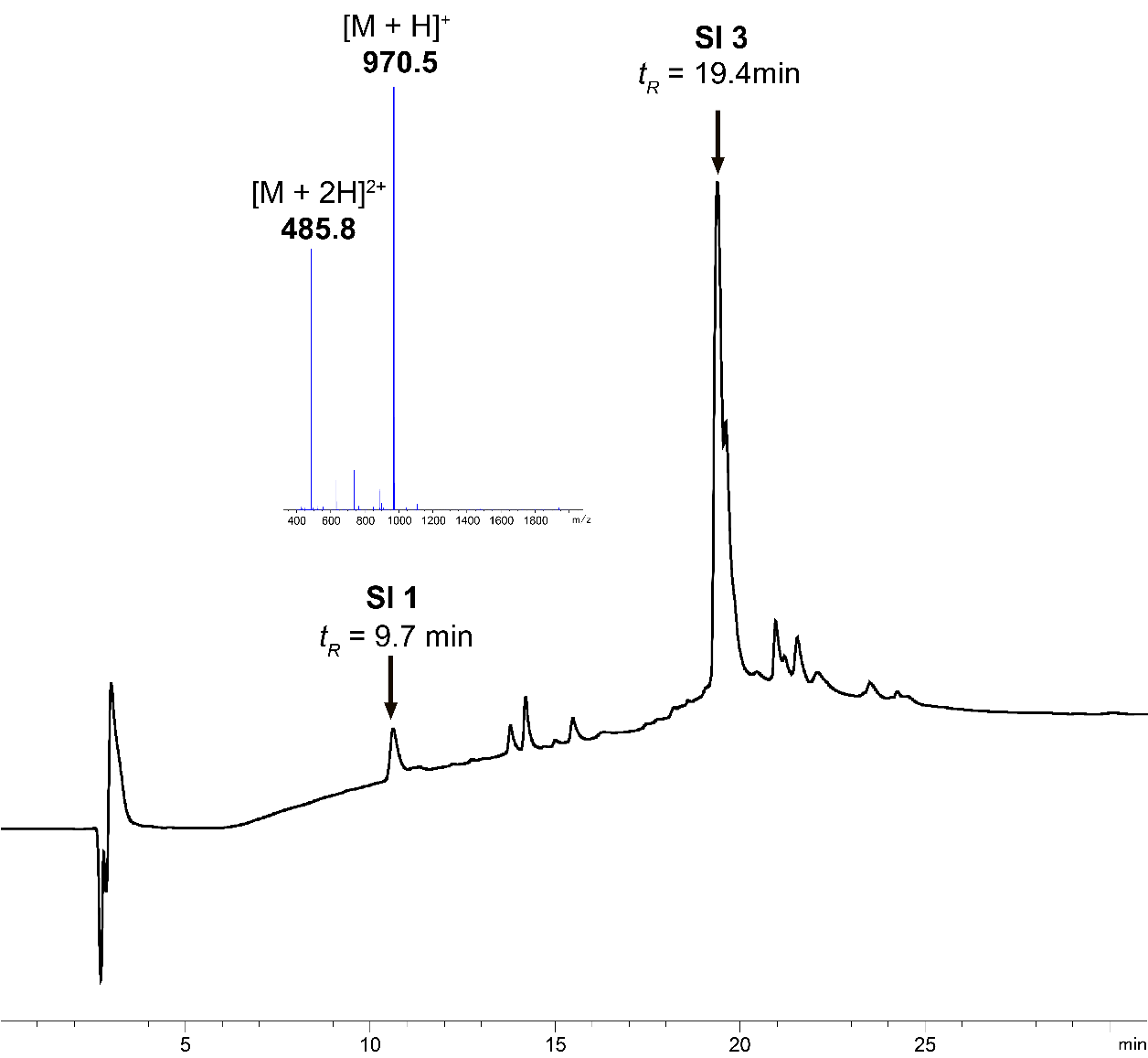


**Figure S2.** LC-MS analysis at 214 nm of branched peptidyl resin **12** represented by **SI 3**.

### Resin cleavage to generate branched depsipeptide **13**.

The *N^α^*-Fmoc protection was removed from resin **12** with one treatment of piperidine/DMF (5 mL, 1:4 *v/v*) for 5 min at r.t. The solution was drained, the resin was washed with DMF (3 × 5 mL) and dried by washing with CH_2_Cl_2_ (3 × 5 mL).

The branched depsipeptide was then cleaved from the resin and lyophilized according to **General Method E** to afford **13** as a white powder. Analysis by LC-MS confirmed the identity of **13** (138.3 mg, 37% yield, 66% purity by peak area); LC-MS:
*t_R_ =* 22.1 min, [M + H]^+^ found 1484.5, [C_82_H_101_N_9_O_13_S_2_ + H]^+^ requires 1484.7, **Figure S3**.

**
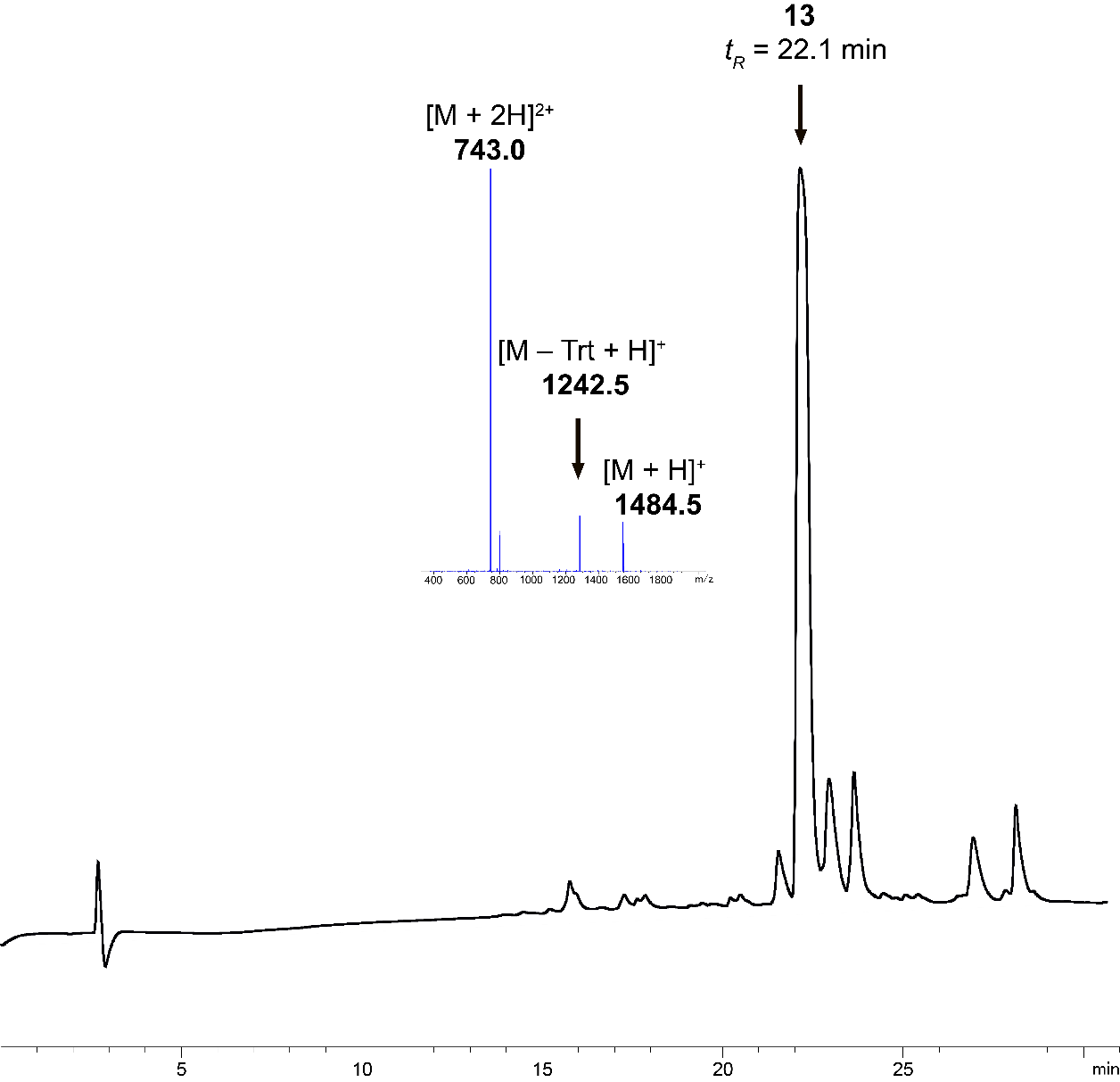
**

**Figure S3.** LC-MS analysis at 214 nm of fully protected cyclization precursor **13**.

### Lactamization and deprotection to cyclic depsipeptide **14**.

Branched depsipeptide **13** (76.6 mg, 0.05 mmol) was cyclized according to **General Method F, i)** to give protected cyclic peptide **SI 4** in 83% conversion (as judged by LC-MS peak area); LC-MS: *t_R_ =* 22.1 min, [ESI-MS, (M – Trt + H)]^+^ found 1224.5, [C_63_H_85_N_9_O_12_S_2_ + H]^+^ requires 1224.6. Side chain deprotection according to **General Method F, ii)** gave peptide **14** as a white fluffy powder (19.3 mg, 51.2% yield, 80% purity) as confirmed by LC-MS: *t_R_ =* 10.6 min, [M + H]^+^ found 730.5, [C_31_H_55_N_9_O_9_S + H]^+^ requires 730.4, **Figure S4**.


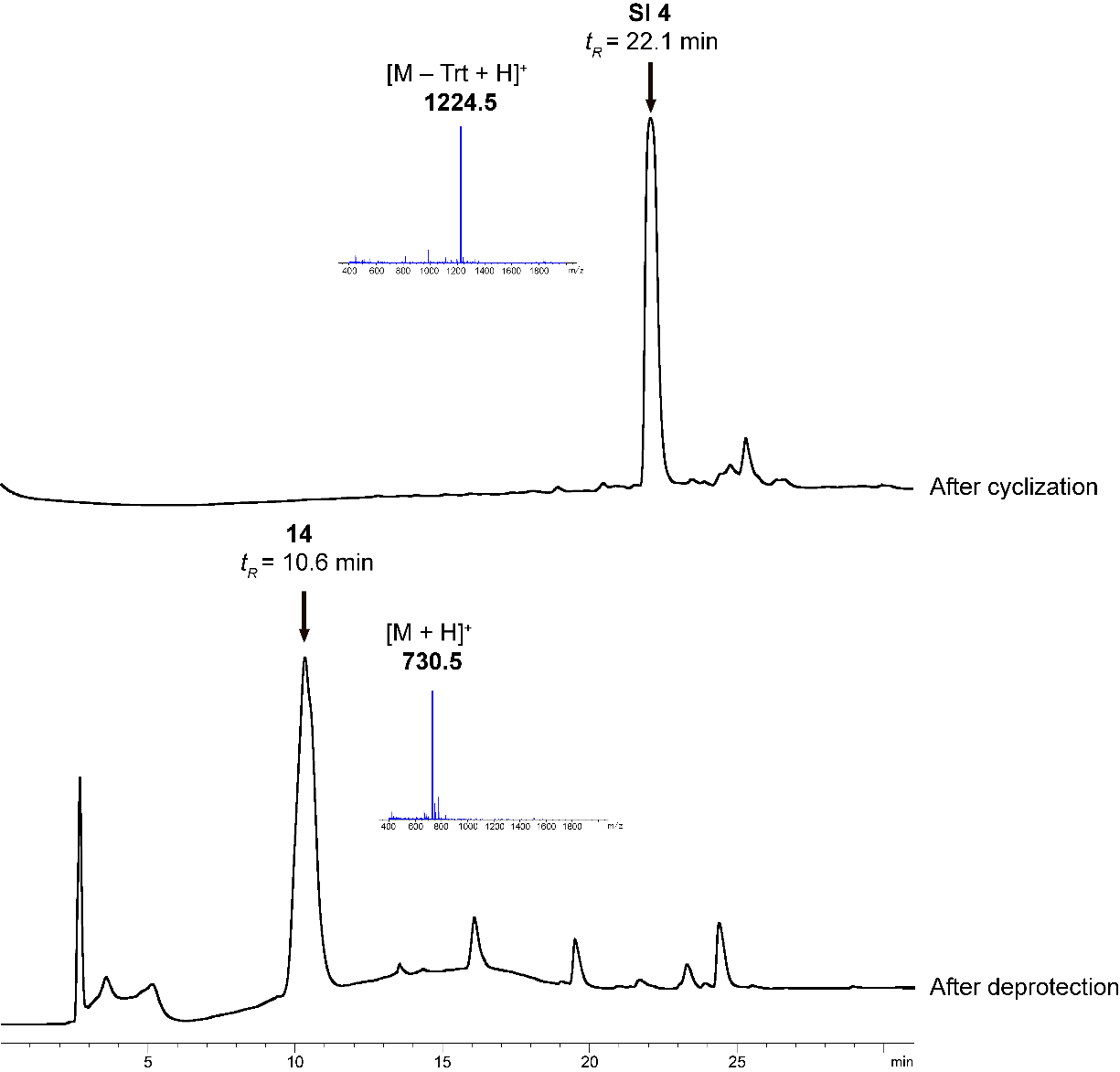


**Figure S4.** LC-MS analysis at 214 nm of side chain protected cyclic product **SI 4** (top) and deprotected cyclic product **14** (bottom).

### Synthesis of truncated S-lipidated teixobactin analog **8a**.

Purified peptide **14** (15.7 mg, 21.5 μmol), DMPA (5.5 mg, 21.5 μmol, 1 eq.), vinyl butyrate (191 μL, 1.5 mmol, 70 eq.) and TIPS (353 μL, 1.7 mmol, 80 eq.) were dissolved in NMP (1.57 mL, 10 mg/mL final concentration w.r.t. **14**) and degassed with argon for 15 min. Volatile liquids *t*NonSH (323 μL, 1.7 mmol, 80 eq.) and TFA (79 μL, 5% *v/v*) were added under argon. The mixture underwent the modified CLipPA reaction according to **General Method G**. Purification according to **General Method H** yielded purified truncated *S-*lipidated teixobactin analog **8a** (2.0 mg, 11.0% yield, 92% purity) as a white solid; RP-HPLC: *t_R_ =* 15.0 min, ESI-MS: [M + H]^+^ found 845.1, [C_37_H_65_N_9_O_11_S + H]^+^ requires 844.5, **Figure S5**.


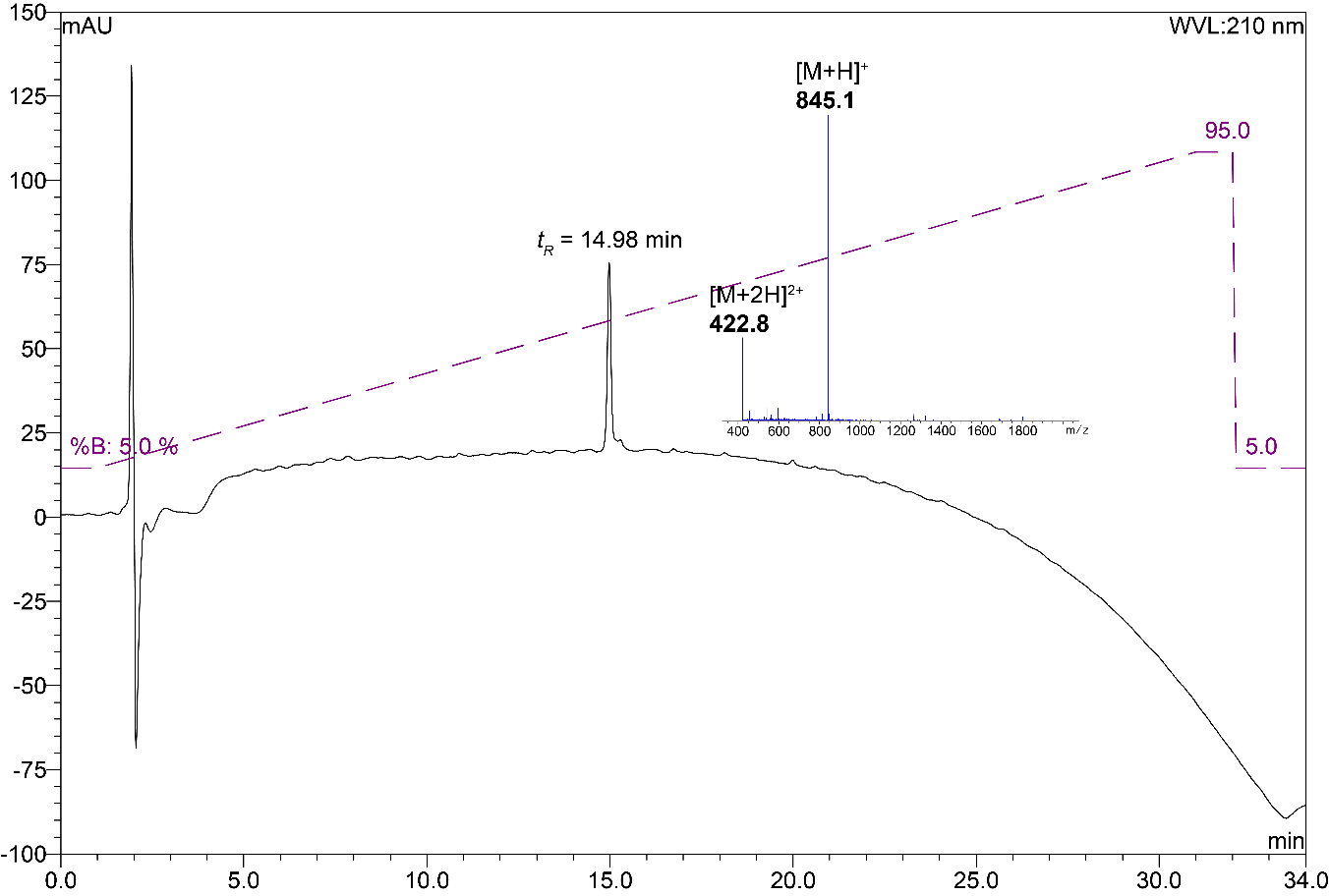


**Figure S5.** Analytical RP-HPLC trace with inset ESI-MS spectrum of truncated *S-*lipidated teixobactin analog **8a** (*ca.* 92% purity as judged by peak area of RP-HPLC at 210 nm); Xterra MS C18 (5 μm 4.6 × 150 mm), linear gradient of 5% B to 95% B over 30 min, *ca.* 3% B per minute at r.t., 1 mL/min.

### Synthesis of truncated S-lipidated teixobactin analog **8b**.

Purified peptide **14** (14.3 mg, 19.6 μmol), DMPA (5.0 mg, 19.6 μmol, 1 eq.), vinyl hexanoate (241 μL, 1.4 mmol, 70 eq.) and TIPS (321 μL, 1.6 mmol, 80 eq.) were dissolved in NMP (1.43 mL, 10 mg/mL final concentration w.r.t. **14**) and degassed with Ar for 15 min. Volatile liquids *t*NonSH (294 μL, 1.6 mmol, 80 eq.) and TFA (72 μL, 5% *v/v*) were added under Ar. The mixture underwent the modified CLipPA reaction according to **General Method G**. Purification according to **General Method H** yielded purified truncated *S-*lipidated teixobactin analog **8b** (2.2 mg, 12.9% yield, 97% purity) as a white solid; RP-HPLC: *t_R_ =* 16.9 min, ESI-MS: [M + H]^+^ found 872.5, [C_39_H_69_N_9_O_11_S + H]^+^ requires 872.5, **Figure S6**.


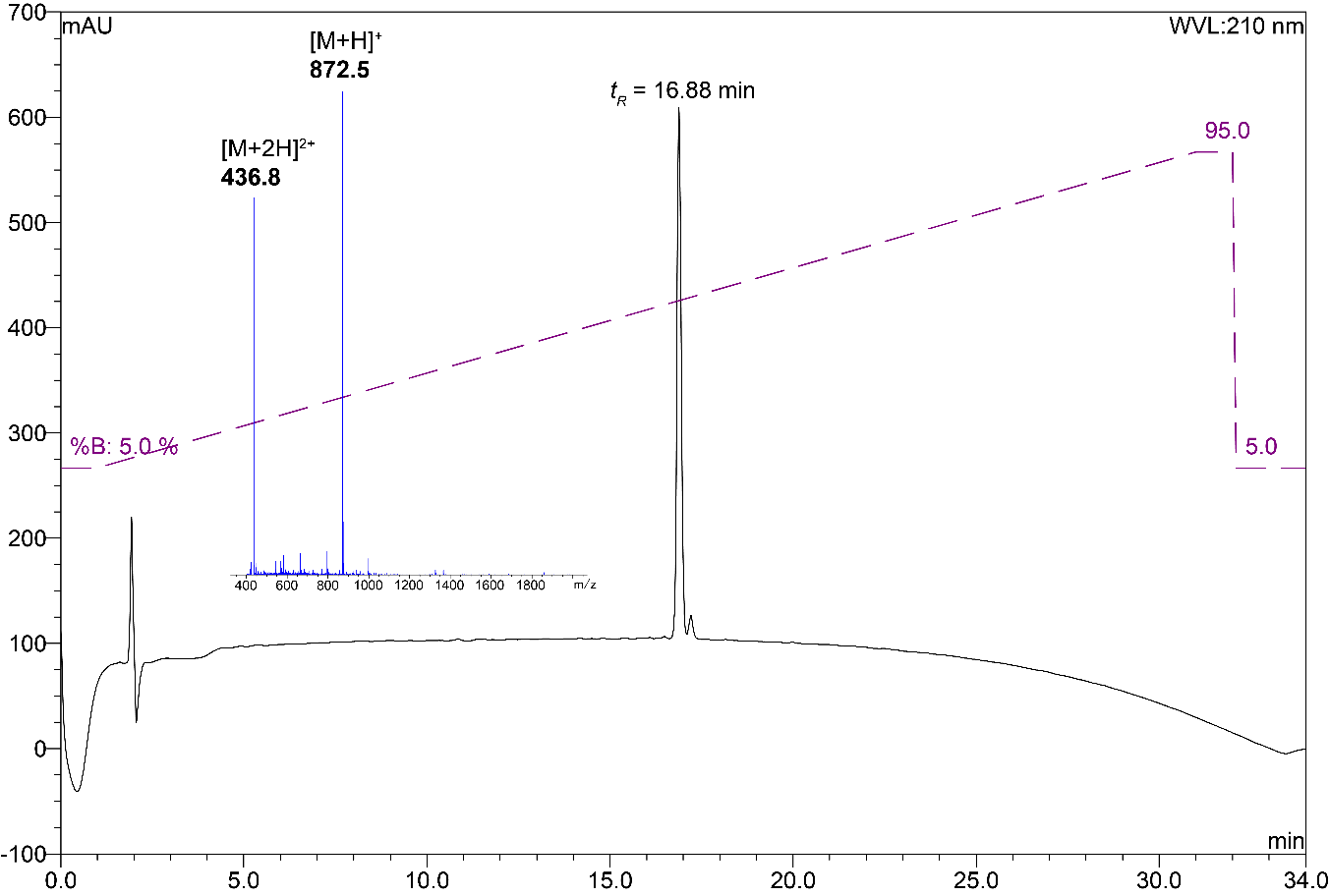


**Figure S6.** Analytical RP-HPLC trace with inset ESI-MS spectrum of truncated *S-*lipidated teixobactin analog **8b** (*ca.* 97% purity as judged by peak area of RP-HPLC at 210 nm); Xterra MS C18 (5 μm 4.6 × 150 mm), linear gradient of 5% B to 95% B over 30 min, *ca.* 3% B per minute at r.t., 1 mL/min.

### Synthesis of truncated S-lipidated teixobactin analog **8c**.

Purified peptide **14** (14.9 mg, 20.4 μmol), DMPA (5.2 mg, 20.4 μmol, 1 eq.), vinyl octanoate (292 μL, 1.4 mmol, 70 eq.) and TIPS (335 μL, 1.6 mmol, 80 eq.) were dissolved in NMP (1.49 mL, 10 mg/mL final concentration w.r.t. **14**) and degassed with Ar for 15 min. Volatile liquids *t*NonSH (306 μL, 1.6 mmol, 80 eq.) and TFA (75 μL, 5% *v/v*) were added under Ar. The mixture underwent the modified CLipPA reaction according to **General Method G**. Purification according to **General Method H** yielded purified truncated *S-*lipidated teixobactin analog **8c** (2.4 mg, 13.1% yield, 97% purity) as a white solid; RP-HPLC: *t_R_ =* 18.9 min, ESI-MS: [M + H]^+^ found 900.6, [C_41_H_73_N_9_O_11_S + H]^+^ requires 900.5, **Figure S7**.


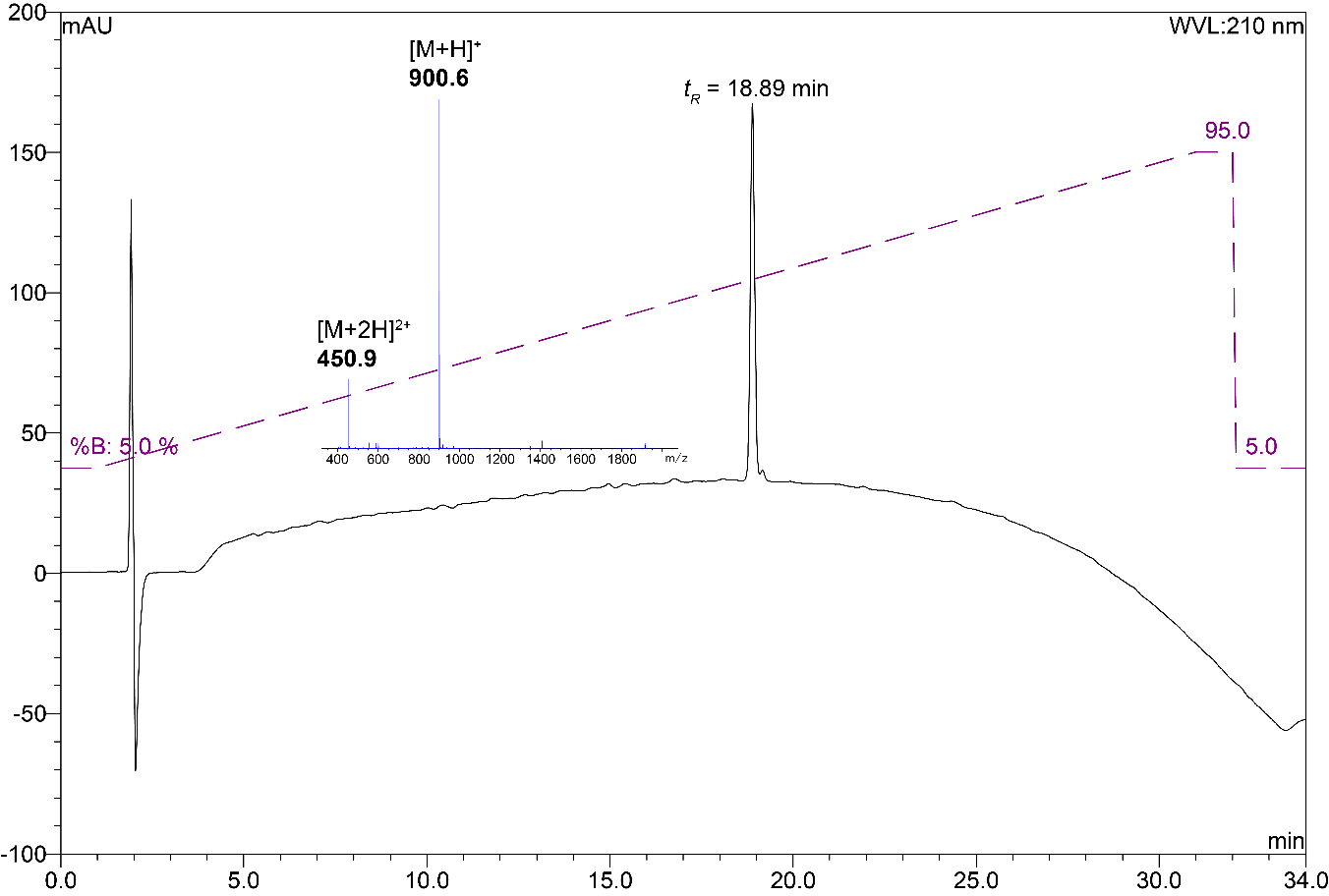


**Figure S7.** Analytical RP-HPLC trace with inset ESI-MS spectrum of truncated *S-*lipidated teixobactin analog **8c** (*ca.* 97% purity as judged by peak area of RP-HPLC at 210 nm); Xterra MS C18 (5 μm 4.6 × 150 mm), linear gradient of 5% B to 95% B over 30 min, *ca.* 3% B per minute at r.t., 1 mL/min.

### Synthesis of truncated S-lipidated teixobactin analog **8d**.

Purified peptide **14** (14.8 mg, 20.3 μmol), DMPA (5.2 mg, 20.3 μmol, 1 eq.), vinyl laurate (357 μL, 1.4 mmol, 70 eq.) and TIPS (333 μL, 1.6 mmol, 80 eq.) were dissolved in NMP (1.48 mL, 10 mg/mL final concentration w.r.t. **14**) and degassed with Ar for 15 min. Volatile liquids *t*NonSH (304 μL, 1.6 mmol, 80 eq.) and TFA (74 μL, 5% *v/v*) were added under Ar. The mixture underwent the modified CLipPA reaction according to **General Method G**. Purification according to **General Method H** yielded purified truncated *S-*lipidated teixobactin analog **8d** (2.3 mg, 11.9% yield, 98% purity) as a white solid; RP-HPLC: *t_R_ =* 23.2 min, ESI-MS: [M + H]^+^ found 956.6, [C_45_H_81_N_9_O_11_S + H]^+^ requires 956.6, **Figure S8**.


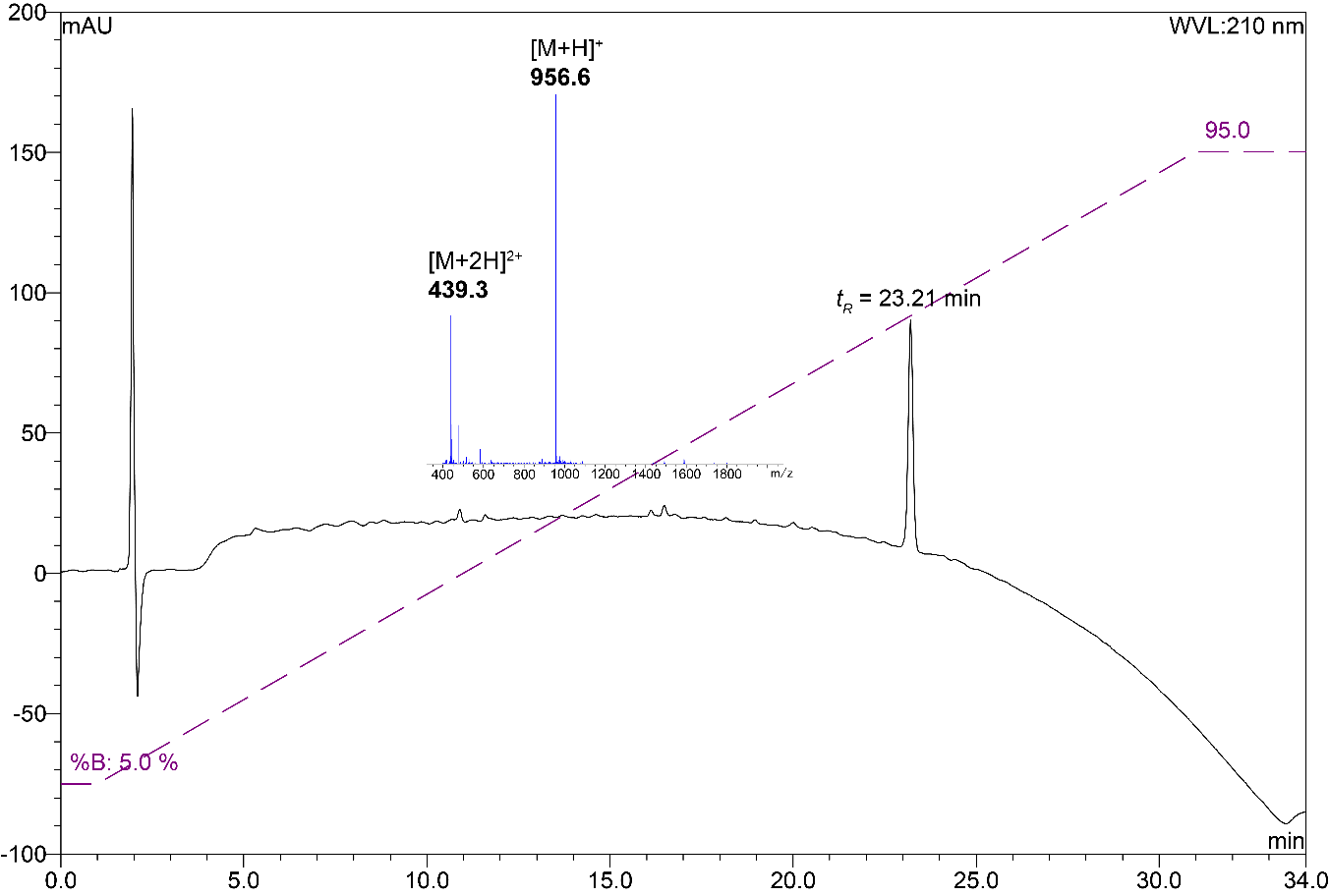


**Figure S8.** Analytical RP-HPLC trace with inset ESI-MS spectrum of truncated *S-*lipidated teixobactin analog **8d** (*ca.* 98% purity as judged by peak area of RP-HPLC at 210 nm); Xterra MS C18 (5 μm 4.6 × 150 mm), linear gradient of 5% B to 95% B over 30 min, *ca.* 3% B per minute at r.t., 1 mL/min.

### Synthesis of truncated S-lipidated teixobactin analog **8e**.

Purified peptide **14** (14.7 mg, 20.2 μmol), DMPA (5.2 mg, 20.2 μmol, 1 eq.), vinyl butyrate (387.6 mg, 1.4 mmol, 70 eq.) and TIPS (330 μL, 1.6 mmol, 80 eq.) were dissolved in NMP (1.47 mL, 10 mg/mL final concentration w.r.t. **14**) and degassed with Ar for 15 min. Volatile liquids *t*NonSH (302 μL, 1.6 mmol, 80 eq.) and TFA (74 μL, 5% *v/v*) were added under Ar. The mixture underwent the modified CLipPA reaction according to **General Method G**. Purification according to **General Method H** yielded purified truncated *S-*lipidated teixobactin analog **8e** (2.1 mg, 10.3% yield, 97% purity) as a white solid; RP-HPLC: *t_R_ =* 28.0 min, ESI-MS: [M + H]^+^ found 1012.6, [C_49_H_89_N_9_O_11_S + H]^+^ requires 1012.6, **Figure S9**.


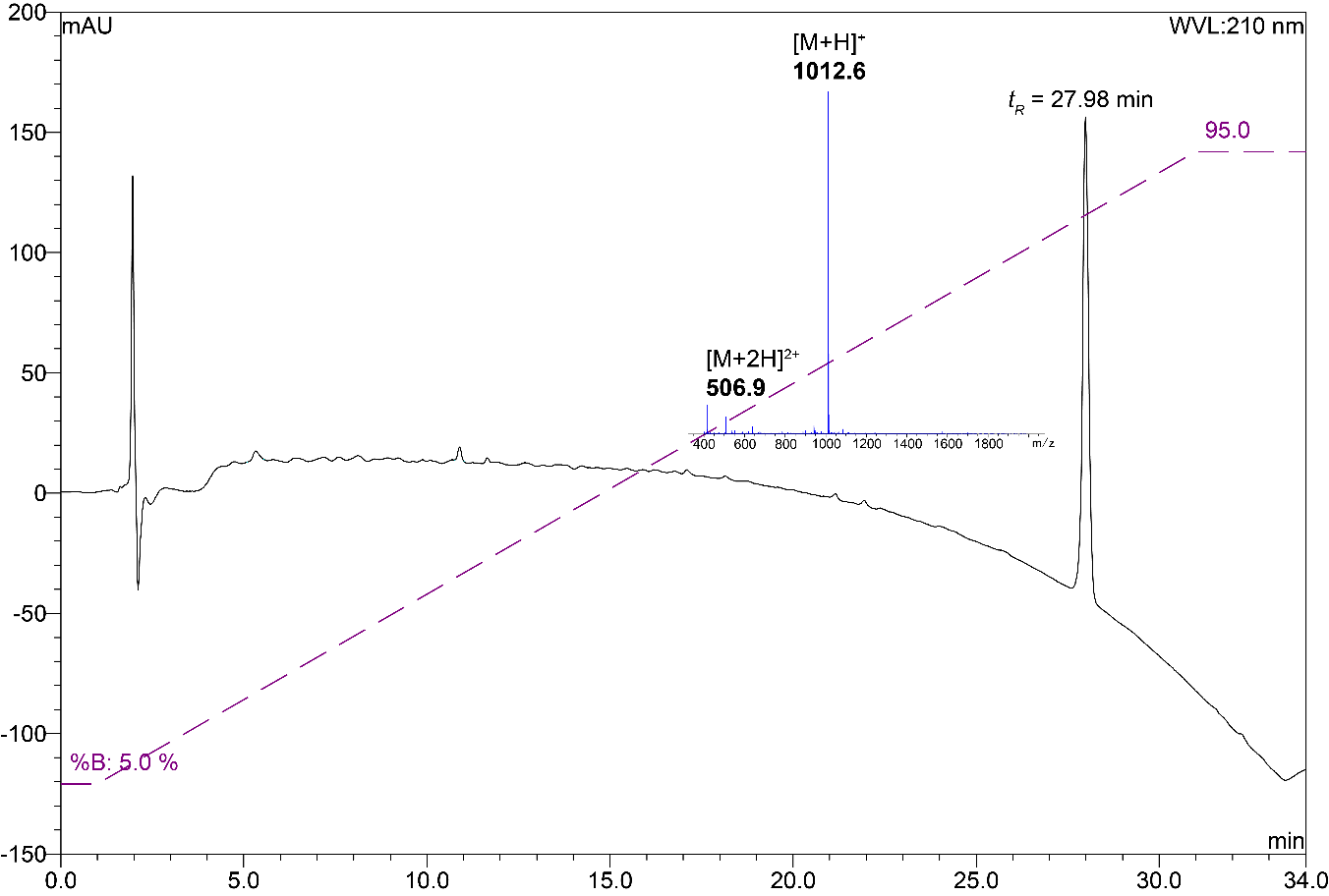


**Figure S9.** Analytical RP-HPLC trace with inset ESI-MS spectrum of truncated *S-*lipidated teixobactin analog **8e** (*ca.* 97% purity as judged by peak area of RP-HPLC at 210 nm); Xterra MS C18 (5 μm 4.6 × 150 mm), linear gradient of 5% B to 95% B over 30 min, *ca.* 3% B per minute at r.t., 1 mL/min.

### Synthesis of truncated S-lipidated teixobactin analog **8f**.

Purified peptide **14** (16.0 mg, 21.9 μmol), DMPA (5.6 mg, 21.9 μmol, 1 eq.), vinyl benzoate (190 μL, 1.5 mmol, 70 eq.) and TIPS (360 μL, 1.8 mmol, 80 eq.) were dissolved in NMP (1.60 mL, 10 mg/mL final concentration w.r.t. **14**) and degassed with Ar for 15 min. Volatile liquids *t*NonSH (329 μL, 1.8 mmol, 80 eq.) and TFA (80 μL, 5% *v/v*) were added under Ar. The mixture underwent the modified CLipPA reaction according to **General Method G**. Purification according to **General Method H** yielded purified truncated *S-*lipidated teixobactin analog **8f** (2.3 mg, 12.0% yield, 93% purity) as a white solid; RP-HPLC: *t_R_ =* 15.9 min, ESI-MS: [M + H]^+^ found 878.5, [C_40_H_63_N_9_O_11_S + H]^+^ requires 878.4, **Figure S10**.


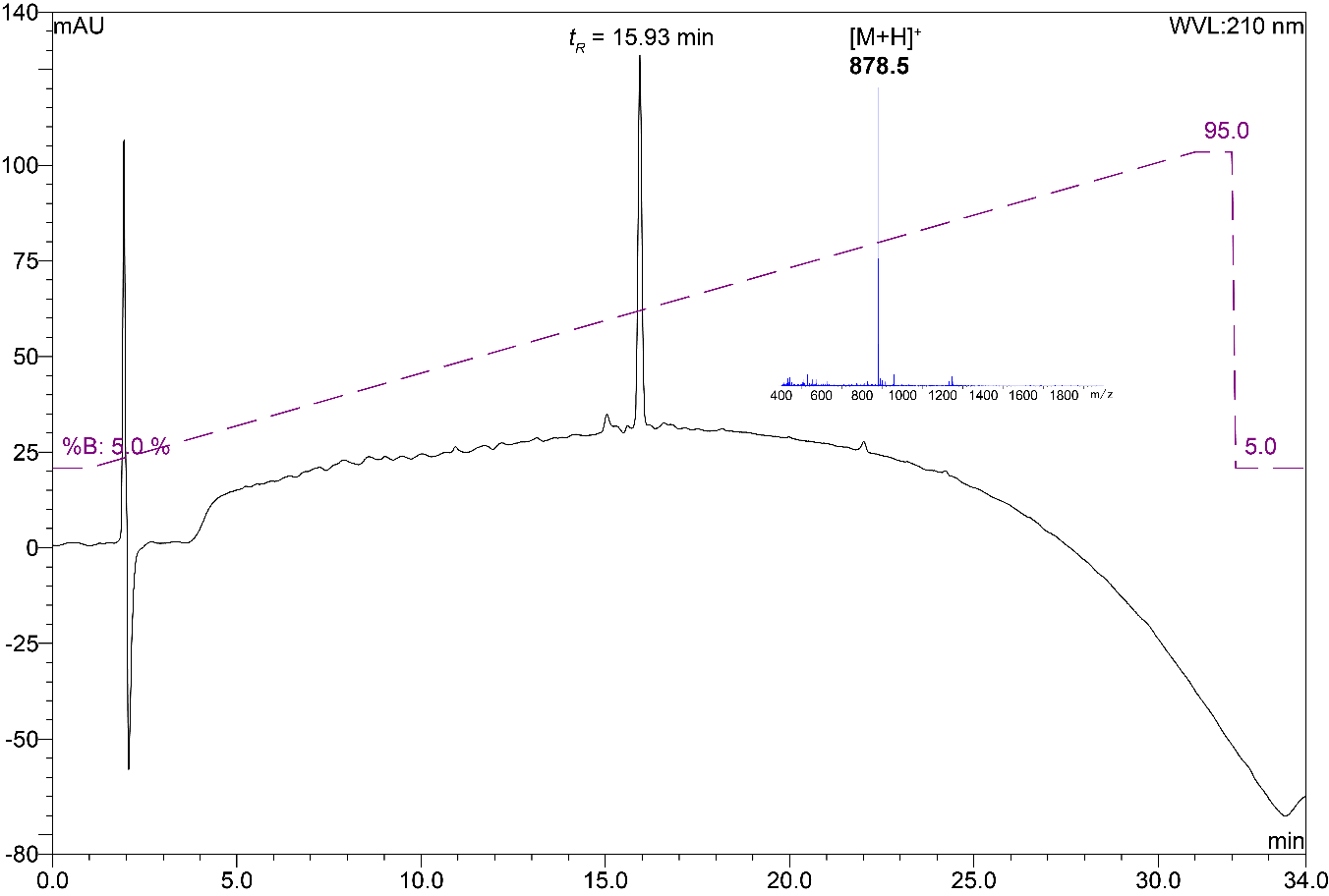


**Figure S10.** Analytical RP-HPLC trace with inset ESI-MS spectrum of truncated *S-*lipidated teixobactin analog **8f** (*ca.* 93% purity as judged by peak area of RP-HPLC at 210 nm); Xterra MS C18 (5 μm 4.6 × 150 mm), linear gradient of 5% B to 95% B over 30 min, *ca.* 3% B per minute at r.t., 1 mL/min.

## Synthesis of lipobactin 3

Ester resin **10** (0.051 mmol, 0.64 mmol/g) was elongated with amino acids (0.20 mmol, 4 eq.) and HATU (74 mg, 0.19 mmol, 3.8 eq.) DIPEA (68 μL, 0.4 mmol, 8 eq.) according to **General Method C** in the order of Fmoc-Ala-OH (64 mg), Fmoc-d-Thr(OH)-OH (70 mg), Fmoc-Ser(*t*Bu)-OH (78 mg), Fmoc-Ile-OH (72 mg), lauric acid (41 mg) to afford peptidyl resin **15**. Following **General Method B,** with the following modifications, Fmoc-Ile-OH (180 mg, 0.51 mmol, 10 eq.) was esterified onto peptidyl resin **11** with DIC (40 μL, 0.26 mmol, 5 eq.) and DMAP (3 mg, 0.026 mmol, 0.5 eq.) to generate the desired branched depsipeptidyl resin. The *N^α^*-Fmoc protection was removed with one treatment of piperidine/DMF (5 mL, 1:4 *v/v*) for 5 min at r.t. The solution was drained, the resin washed with DMF (3 × 5 mL) and dried by washing with CH_2_Cl_2_ (3 × 5 mL). The desired side chain protected branched depsipeptide was then liberated from the resin and lyophilized according to **General Method E.** The branched peptide was cyclized and side chain protecting groups removed according to **General Method F**. The crude cyclic peptide was purified according to **General Method H**, affording lipobactin **3** (15.1 mg, 32.7% yield, 95% purity) as a flaky white powder; RP-HPLC: *t_R_ =* 20.4 min, ESI-MS: [M + H]^+^ found 824.6, [C_41_H_75_N_9_O_9_ + H]^+^ requires 824.6, **Figure S11**.


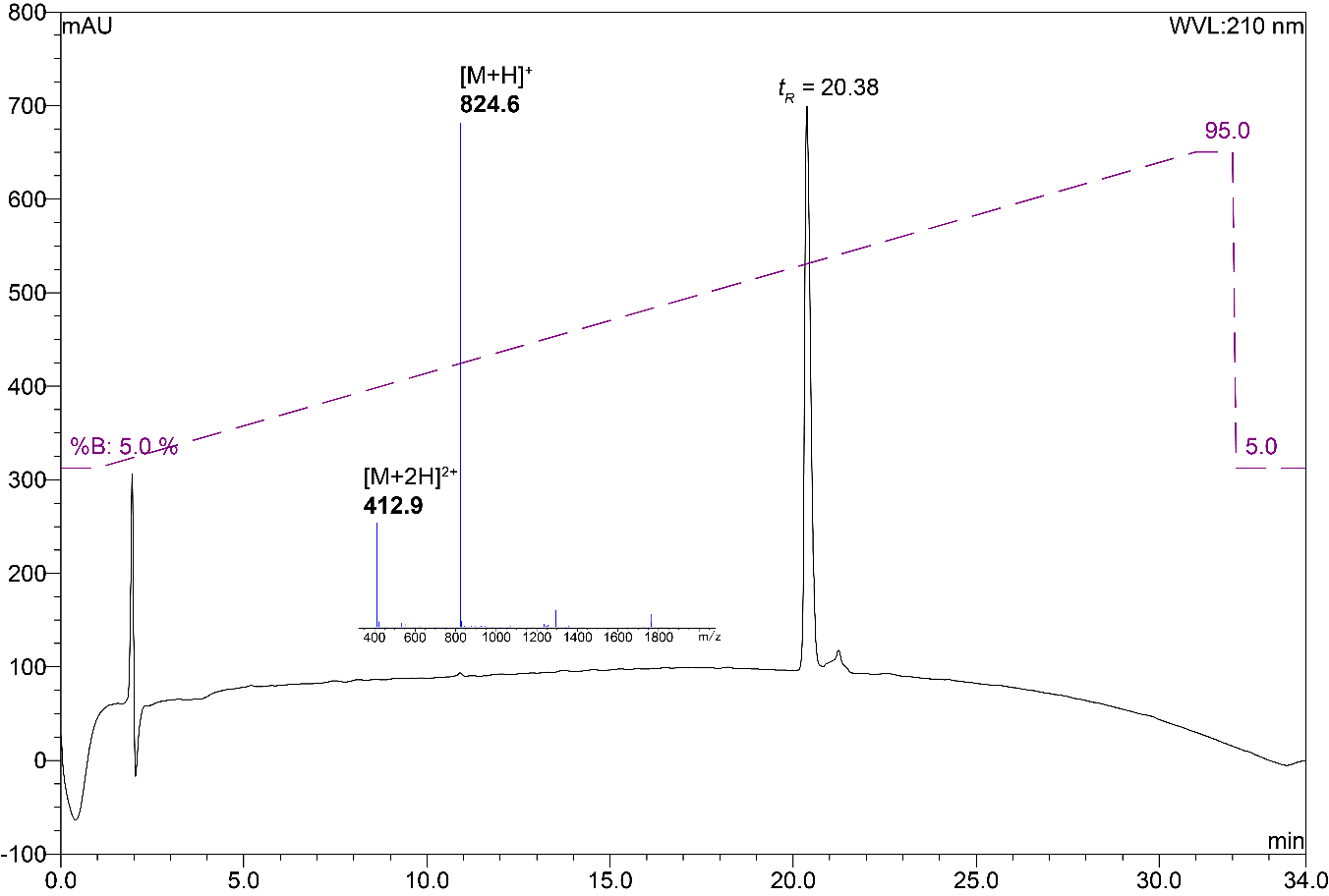


**Figure S11.** Analytical RP-HPLC trace with inset ESI-MS spectrum of lipobactin **3** (*ca.* 95% purity as judged by peak area of RP-HPLC at 210 nm); Xterra MS C18 (5 μm 4.6 × 150 mm), linear gradient of 5% B to 95% B over 30 min, *ca.* 3% B per minute at r.t., 1 mL/min.

# Antibacterial susceptibility testing

*Staphylococcus aureus* ATCC 29213 was grown in non-cation adjusted Mueller Hinton (MH) broth at 37 °C with shaking (200 rpm). MIC assay was performed according to a literature protocol.^5^ Briefly**,** a two-fold dilution series (from 128 μM to 0.0625 μM, final) was prepared in polypropylene 96-well plates using MH media. Overnight cultures of bacteria were diluted in fresh MH before adding 50 μL of inoculum to each well of the MIC plate, to achieve a final volume of 100 μL with a uniform CFU/ml of ~5 x10^5^ in each well. Plates were incubated at 37 °C with shaking for 24 h before determining the MIC. MIC’s were determined as the lowest concentration at which growth did not occur (**Table S1**).

**Table S1.** MIC values of lipobactin **3** and truncated *S-*lipidated teixobactin analogs **8a** – **8f** in μM.

| Peptide | *S. aureus* ATCC 29213 |
| --- | --- |
| **3** | 8 |
| **8a** | > 128 |
| **8b** | > 128 |
| **8c** | > 128 |
| **8d** | > 128 |
| **8e** | > 128 |
| **8f** | > 128 |

# References.

(1) Kaiser, E.; Colescott, R. L.; Bossinger, C. D.; Cook, P. I. Color Test for Detection of Free Terminal Amino Groups in the Solid-Phase Synthesis of Peptides. *Anal. Biochem.* **1970**, *34* (2), 595–598. https://doi.org/10.1016/0003-2697(70)90146-6.

(2) Chan, W. C.; White, P. D. Basic Procedures. In *Fmoc solid phase peptide synthesis: a practical approach*; Chan, W. C., White, P. D., Eds.; Oxford University Press, 2000; pp 41–76.

(3) Harris, P. W. R.; Lee, D. J.; Brimble, M. A. A Slow Gradient Approach for the Purification of Synthetic Polypeptides by Reversed Phase High Performance Liquid Chromatography. *J. Pept. Sci.* **2012**, *18* (9), 549–555. https://doi.org/10.1002/psc.2432.

(4) Yang, H.; Chen, K. H.; Nowick, J. S. Elucidation of the Teixobactin Pharmacophore. *ACS Chem. Biol.* **2016**, *11* (7), 1823–1826. https://doi.org/10.1021/acschembio.6b00295.

(5) Wiegand, I.; Hilpert, K.; Hancock, R. E. W. Agar and Broth Dilution Methods to Determine the Minimal Inhibitory Concentration (MIC) of Antimicrobial Substances. *Nat. Protoc.* **2008**, *3*, 163. https://doi.org/10.1038/nprot.2007.521.
